# Reversible acute-on-chronic kidney injury complicating antibiotic dosing in group A streptococcal necrotizing fasciitis at a remote Indonesian hospital: a case report

**DOI:** 10.1093/jscr/rjag808

**Published:** 2026-09-20

**Authors:** Zikri Aulio Septiawan, Nabila Alsya Dwi Nirvana, Indra Kasman, I Komang Agus Setiawan

**Affiliations:** Department of Surgery, dr. T.C. Hillers Regional General Hospital, Maumere, Sikka Regency, East Nusa Tenggara, Indonesia; Department of Internal Medicine, dr. T.C. Hillers Regional General Hospital, Maumere, Sikka Regency, East Nusa Tenggara, Indonesia; Department of Internal Medicine, dr. T.C. Hillers Regional General Hospital, Maumere, Sikka Regency, East Nusa Tenggara, Indonesia; Department of Surgery, dr. T.C. Hillers Regional General Hospital, Maumere, Sikka Regency, East Nusa Tenggara, Indonesia

**Keywords:** necrotizing fasciitis, *Streptococcus pyogenes*, acute kidney injury, chronic kidney disease, pharmacokinetics, anti-bacterial agents

## Abstract

Necrotizing fasciitis is a surgical emergency in which delayed source control increases mortality, and concurrent severe renal impairment both disrupts antibiotic dosing and risks misclassification as irreversible failure. A 72-year-old woman presented to a remote Indonesian district hospital with 5 days of right lower-leg pain, swelling, bullae and purulent discharge after a clinic incision. Admission creatinine was 4.6 mg/dl, rising to 5.0 mg/dl (estimated glomerular filtration rate ~9), with leukocytosis; ultrasonography showed small echogenic kidneys indicating chronic kidney disease. Initially treated as bullous cellulitis, the lesion progressed; a pre-operative wound culture grew *Streptococcus pyogenes*. Creatinine recovered to 1.17 mg/dl by postoperative Day 4, identifying severe but reversible acute-on-chronic injury. Antibiotics dosed to the acute nadir risked relative under-dosing as function recovered. An acutely elevated creatinine during sepsis can overstate the true baseline; serial measurement is essential before labelling end-stage disease or anchoring antibiotic dosing to the nadir.

## Introduction

Necrotizing fasciitis (NF) is a fulminant soft-tissue infection in which necrosis outpaces the overlying skin; contemporary mortality is ~23%, rising with delayed debridement [1]. Management rests on early source control [2] and broad-spectrum antibiotics including an anti-exotoxin agent [3]. In remote archipelagic Indonesian hospitals classified as DTPK (*Daerah Terpencil, Perbatasan, dan Kepulauan —* remote, border and island areas), C-reactive protein, lactate, cross-sectional imaging, therapeutic drug monitoring and rapid inter-island transfer are frequently unavailable. Severe renal impairment compounds care: it disrupts the pharmacokinetics of renally cleared antimicrobials and, when first found during acute illness, is easily misclassified. Most Indonesian chronic kidney disease (CKD) is undiagnosed at presentation [4]. We report a case combining an atypical host, a misleading renal snapshot and a constrained system, reported in line with the Surgical CAse REport (SCARE) 2023 guideline [5].

## Case report

A 72-year-old woman (53 kg, 140 cm) presented to a remote district hospital in eastern Indonesia with 5 days of right lower-leg pain and swelling and 1 day of blood-tinged purulent discharge. The illness had begun a week earlier with fever and erythema, then a boil-like lesion incised at a primary clinic, after which it enlarged. Her only comorbidity was gout, treated with allopurinol. She had also been started on trimethoprim-sulfamethoxazole and paracetamol at the primary clinic a week earlier, presumably as empiric cover for the soft-tissue infection; she had no known diabetes, hypertension or kidney disease and no prior creatinine on record.

She was alert (blood pressure 100/60 mmHg, heart rate 97/min, temperature 37.4°C). The right cruris showed diffuse erythema and oedema with a central violaceous plaque (~8 × 6 cm), tense serosanguineous bullae and focal ulceration discharging pus (Fig. 1A); pain was disproportionate to the visible inflammation. White cells were 35 790/μl, haemoglobin 10.1 g/dl, creatinine 4.6 mg/dl [rising to 5.0; estimated glomerular filtration rate (eGFR) ~9 ml/min/1.73 m^2^] and urea 181 mg/dl; C-reactive protein and lactate were unavailable. A partial laboratory risk indicator for necrotizing fasciitis (LRINEC) score was 6, but the creatinine criterion is triggered by any cause of renal impairment, mechanically inflating it [6]. Ultrasonography showed bilateral small, echogenic kidneys with loss of corticomedullary differentiation — a chronic pattern indicating underlying CKD.

**Figure 1:**
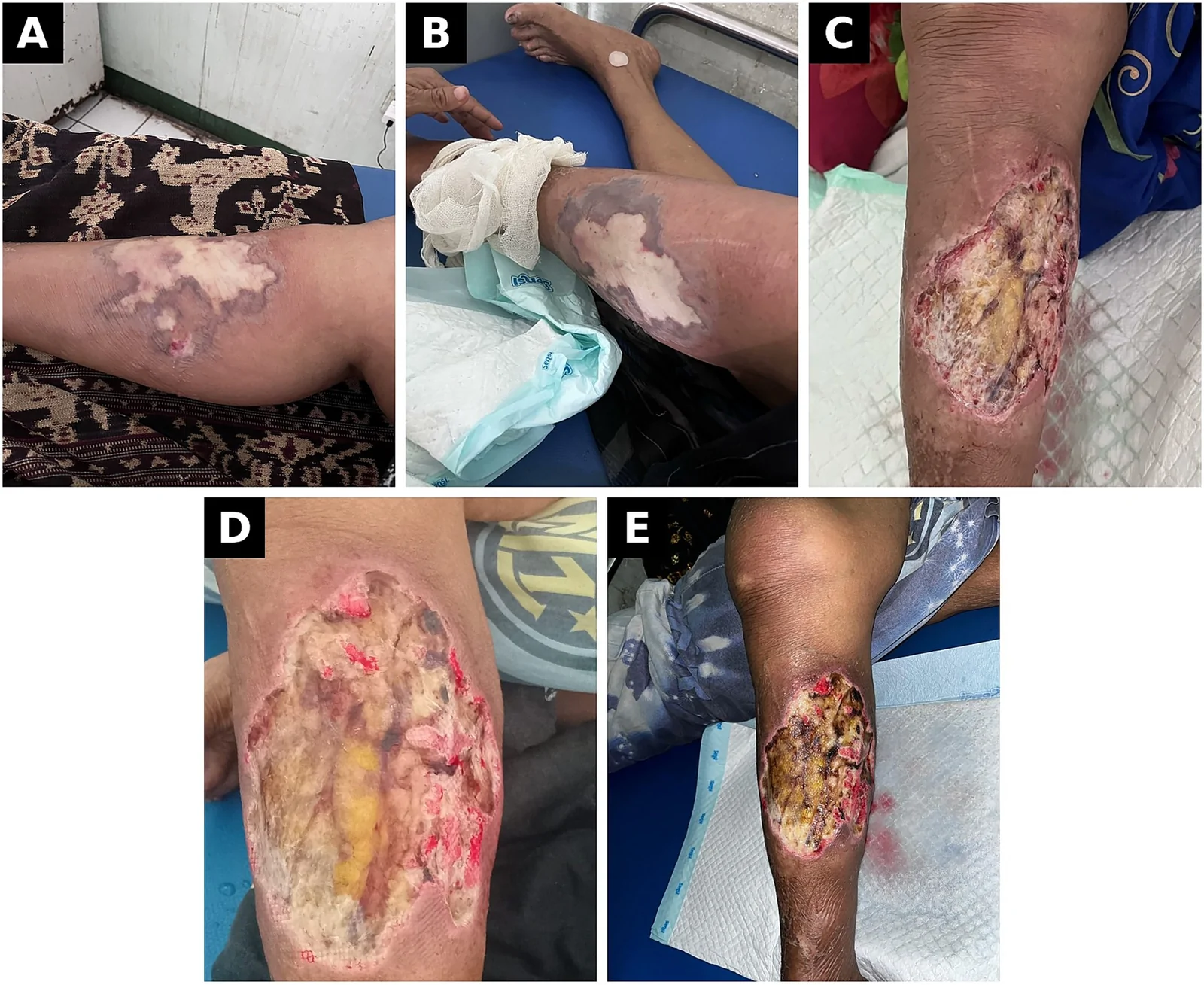
Clinical course of right-cruris necrotizing fasciitis. (A) At presentation: A central pale, devitalized plaque with surrounding violaceous discoloration and a focal ulcerated point at the site of an earlier primary-clinic incision, the appearance initially interpreted as bullous cellulitis. (B) After 5 days of ceftriaxone, before debridement: Progression to a confluent pale-white eschar with surrounding violaceous skin and tense oedema. (C) Postoperative Day 1: The surgical defect after excision to healthy bleeding margins, with viable preserved muscle. (D) Postoperative Day 4: Early beefy-red granulation; yellow areas are residual tulle dressing, not infection. (E) Postoperative Day 6: Further granulation with margin contraction and no recurrent necrosis.

Over 5 days of ceftriaxone the lesion progressed, the violaceous area becoming a pale-white eschar with new proximal bullae (Fig. 1B); meropenem replaced ceftriaxone [7]. Inter-facility transfer (nearest tertiary centre ~24 h away by sea ferry) was infeasible. After written family consent, debridement was performed on hospital Day 7 (ASA-PS III) under spinal anaesthesia (hyperbaric bupivacaine 7.5 mg with fentanyl 25 μg) [8]; the on-duty general practitioner assisted under the consultant surgeon’s direct supervision. Intra-operative findings — dishwater exudate, grey friable fat and avascular fascia separating on digital probing (positive finger test) [9] — confirmed NF. A wound swab culture, sent on hospital Day 3, became available on the day of debridement and grew *Streptococcus pyogenes*, susceptible to ciprofloxacin, clindamycin, erythromycin, trimethoprim-sulfamethoxazole and vancomycin.

Postoperatively, the surgical defect demonstrated viable muscle and healthy margins (Fig. 1C). IV levofloxacin (500 mg loading, then 250 mg every 48 h) was started, guided by regimens validated in end-stage renal disease [10] and population data in older adults [11]. Oral clindamycin 300 mg every 8 h was added as an anti-exotoxin agent; intravenous clindamycin (the IDSA-recommended 600–900 mg every 8 h) [3] was not on the formulary, so the oral route (bioavailability ~87.6%) [12] was used despite a sub-guideline dose, given source control and a susceptible isolate. Creatinine recovered to 1.17 mg/dl (eGFR ~50; CKD stage 3a) by postoperative Day 4, identifying severe but largely reversible sepsis-associated acute kidney injury on occult CKD — attributable to sepsis, hypovolaemia and nephrotoxic exposure [a brief nonsteroidal anti-inflammatory drug (NSAID) course and trimethoprim, which also inhibits tubular creatinine secretion] [13]. Dialysis was never required. Leukocytosis normalized to ~8000/μl within 72 h; haemoglobin fell to 7.1 g/dl (transfused to 9.1 g/dl). The wound granulated steadily (Fig. 1D and E), and she was discharged on hospital Day 14, remaining well at outpatient review. The timeline is summarized in Table 1.

**Table 1:** Timeline of key events

Hospital day	Event	Action
1	Pain, bullae, pus (Fig. 1A); WBC 35 790/μl; creatinine 4.6 mg/dl (eGFR ~9)	Admitted as bullous cellulitis; IV ceftriaxone
3	Creatinine 5.0 mg/dl; lesion unchanged	Ceftriaxone continued; renal ultrasound ordered; wound swab sent
5	Pale-white eschar, new bullae (Fig. 1B)	NF suspected; switched to renally adjusted meropenem
6	Small echogenic kidneys on ultrasound; transfer infeasible	Family consent for debridement obtained
7–8 (POD 0–1)	Operative debridement; positive finger test; viable muscle and healthy margins (Fig. 1C); culture result available – *Streptococcus pyogenes*, clindamycin-susceptible	Meropenem stopped; levofloxacin + oral clindamycin started, guided by the isolate
11 (POD 4)	Creatinine 1.17 mg/dl (eGFR ~50); WBC ~8000/μl; granulation (Fig. 1D)	Renal recovery confirmed; transfusion for anaemia
13–14 (POD 6–7)	Wound granulating (Fig. 1E); haemoglobin 9.1 g/dl	Discharged on oral levofloxacin and clindamycin

WBC, white blood cell count; eGFR, estimated glomerular filtration rate; NF, necrotizing fasciitis; POD, postoperative day.

## Discussion

The instructive feature is the renal trajectory. An admission creatinine of 4.6–5.0 mg/dl in a frail elderly woman invited a label of end-stage renal disease, yet recovery to 1.17 mg/dl within days of source control identified reversible acute-on-chronic injury [13]. Small echogenic kidneys establish chronicity but not stage; trimethoprim further raises measured creatinine without an equivalent fall in true filtration. An isolated, acutely elevated creatinine during sepsis should not by itself establish irreversible disease — serial measurement after resuscitation and source control is essential before counselling toward dialysis or anchoring long-term therapy to the acute value.

This carries a pharmacological corollary. Renally cleared antimicrobials dosed to the nadir are progressively under-dosed if function recovers: the levofloxacin interval was anchored to an eGFR of ~9, yet by Day 4 the eGFR had reached ~50 — a five-fold change reducing exposure relative to target [11]. Where therapeutic drug monitoring is unavailable, renal function should be re-estimated at least daily during recovery and intervals shortened, while recognizing that source control is the principal determinant of outcome in NF [1, 2].

Adjunctive clindamycin is recommended for invasive group A streptococcal infection because it suppresses exotoxin production independently of growth phase, with a demonstrated mortality benefit [3, 14]; our sub-guideline oral dose reflected a formulary gap that district hospitals should close. The LRINEC score should be treated as supportive only — two of six variables were unavailable, the creatinine criterion was inflated by renal impairment, and its pooled sensitivity for extremity NF is modest [6] — so a low score must never delay operative exploration. Limitations include the single-patient design, absent C-reactive protein, lactate, drug-level monitoring and any prior creatinine to define the true CKD baseline, sparse local susceptibility data, and the absence of a formal patient-perspective statement.

In summary, an admission creatinine in the ‘stage 5’ range proved to be severe but reversible acute-on-chronic injury that recovered to CKD stage 3a. Clinicians should obtain a baseline creatinine in every elderly patient presenting acutely, avoid premature labelling of irreversible disease, dose antibiotics to a renal function re-estimated as it recovers, and ensure intravenous anti-exotoxin agents are stocked where streptococcal soft-tissue infection is treated.

## Data Availability

The data supporting this report are contained within the article. The underlying clinical records contain identifiable information and are not publicly available to protect patient privacy, but de-identified data may be provided by the corresponding author on reasonable request, subject to institutional approval.
